# Supplementary material for: Differentially expressed genes reflect disease-induced rather than disease-causing changes in the transcriptome
Source: Nat Commun. 2021 Sep 24;12:5647. doi: 10.1038/s41467-021-25805-y (PMC8463674; doi:10.1038/s41467-021-25805-y)
Supplement: Supplementary file 1 — Supplementary Information File [file 41467_2021_25805_MOESM1_ESM.pdf]

Supplementary Information

Differentially expressed genes reflect disease-induced rather than disease-causing changes in the transcriptome

Porcu et al.

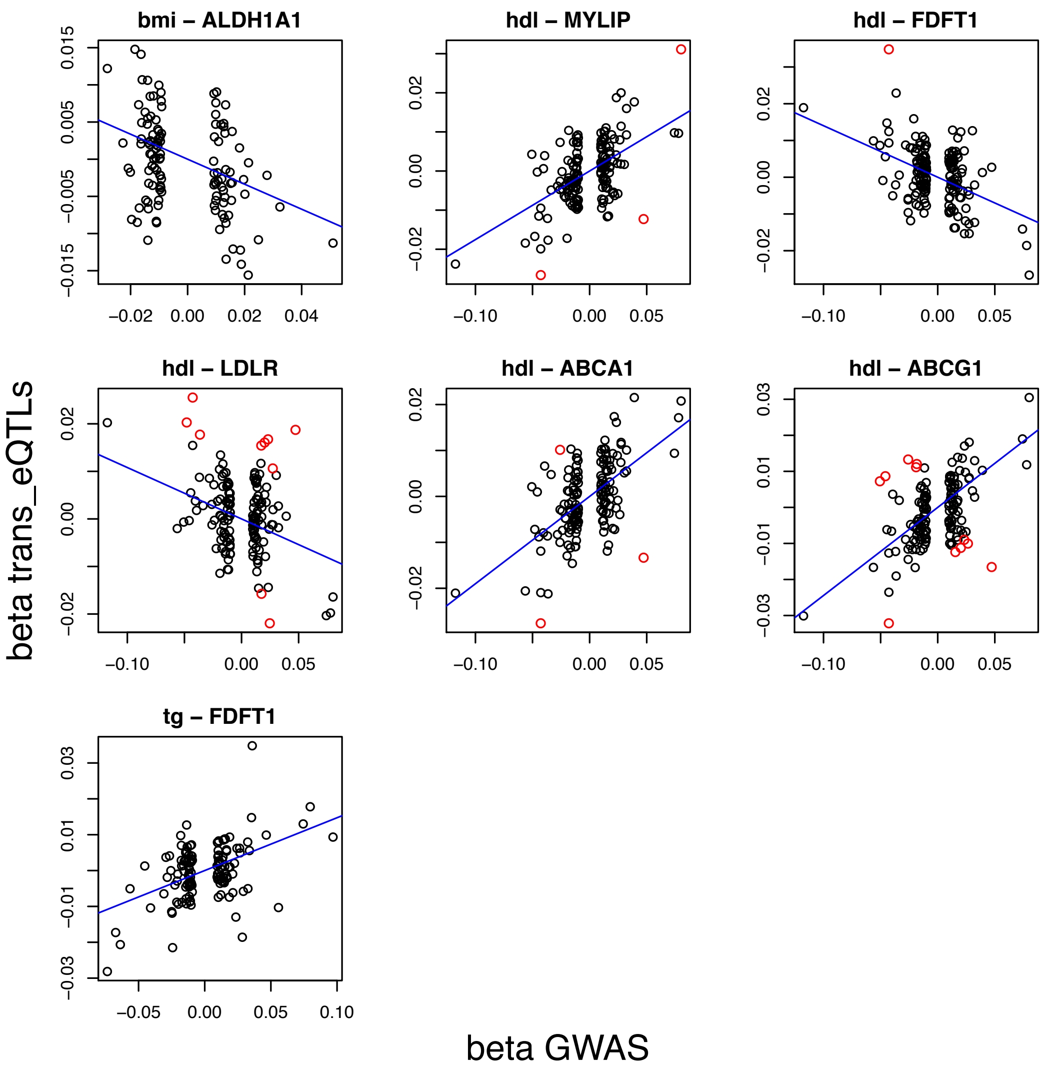

Supplementary Figure 1. ***Scatterplots of significant genes***. *For each SNP we plotted its effect on the phenotype (x-axis, beta GWAS) and on the expression of the gene (y-axis, beta trans eQTLs). The blue line represent the regression line estimated by revTWMR. The red dots indicate the SNPs detected as outliers by the heterogeneity test. Source data are provided as a Source Data file.*

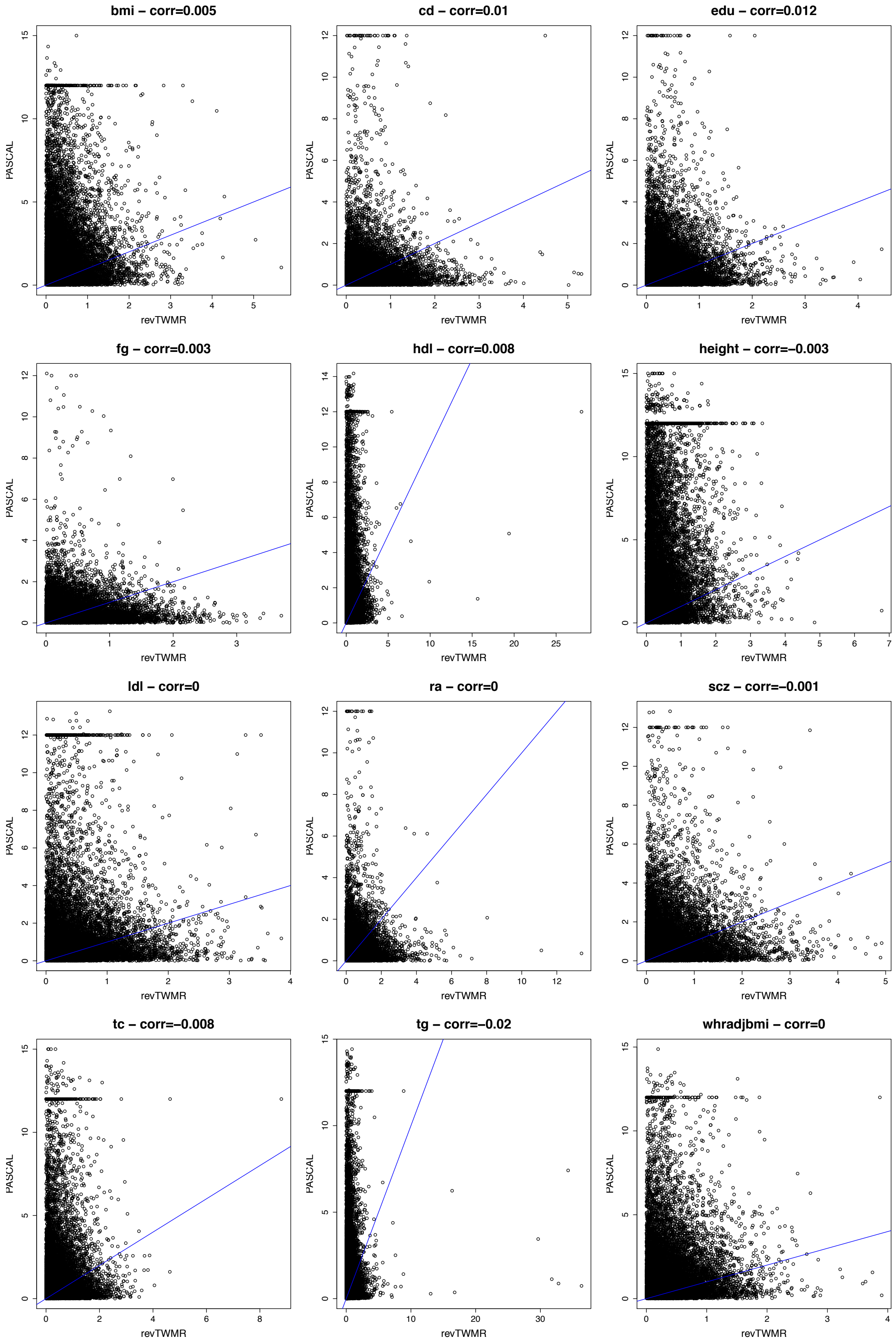

Supplementary Figure 2. ***Comparison between revTWMR and PASCAL results***. *For each gene we compared the –$\log_{10}$(P-value) calculated by revTWMR (x-axis) and PASCAL (y-axis). Source data are provided as a Source Data file.*

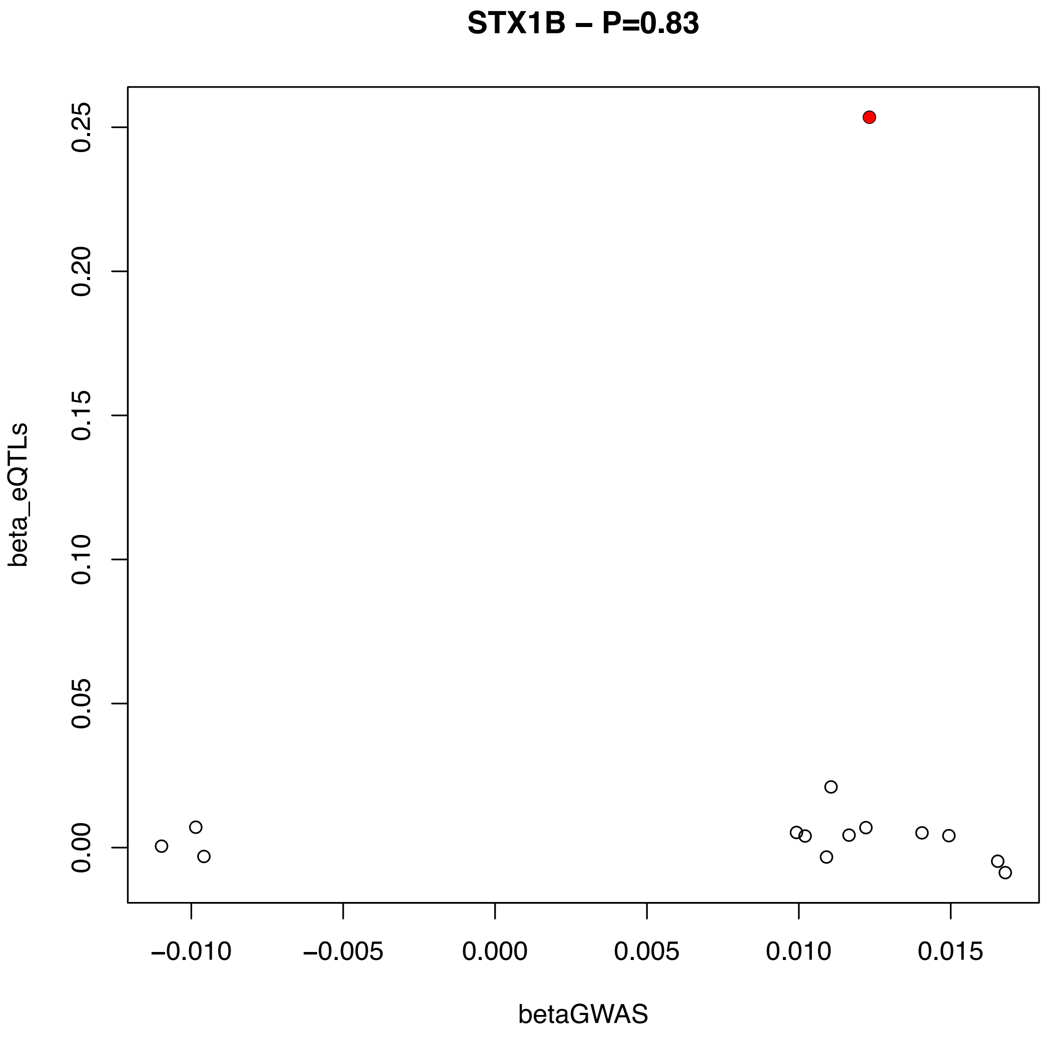

Supplementary Figure 3. ***Pleiotropic SNP leads to biased association for STX1B***. *For each SNP we plotted its effect on educational attainment (x-axis) and on the expression of STX1B (y-axis). The red dot represents rs2456973. Source data are provided as a Source Data file.*

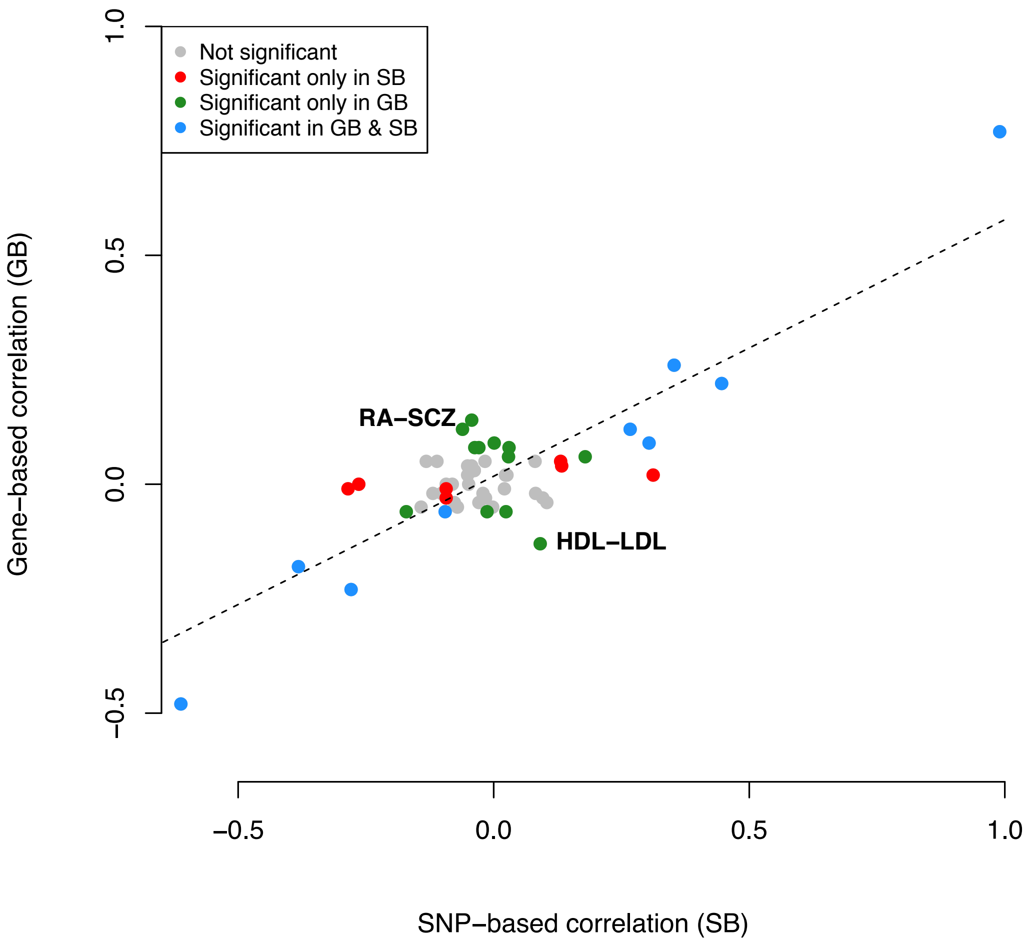

Supplementary Figure 4. ***Linear relationship between the perturbation correlation ($\hat{\rho}_P$) and the genetic correlation ($\hat{\rho}_G$) obtained from LD Score Regression***. *We selected the traits analyzed by our study and Bulik-Sullivan et al [1] and for each pair of traits we compared the two correlations. Gray dots represent non-significant trait pairs, blue dots represent trait pairs significant for both correlation and red and green ones correspond to pairs of traits significant only in $\hat{\rho}_G$ or $\hat{\rho}_P$, respectively. The dotted line represents the regression line. Source data are provided as a Source Data file.*

Supplementary References

1. Bulik-Sullivan, B., et al., *An atlas of genetic correlations across human diseases and traits.* Nat Genet, 2015. **47**(11): p. 1236-41.
