# Supplementary material for: Differentially expressed genes reflect disease-induced rather than disease-causing changes in the transcriptome
Source: Nat Commun. 2021 Sep 24;12:5647. doi: 10.1038/s41467-021-25805-y (PMC8463674; doi:10.1038/s41467-021-25805-y)
Supplement: Supplementary file 3 — Description of Additional Supplementary Files [file 41467_2021_25805_MOESM3_ESM.pdf]

Description of Additional Supplementary Files

File Name: Supplementary Data 1
Description: Significant trait-gene associations

File Name: Supplementary Data 2
Description: Significant gene-trait associations

File Name: Supplementary Data 3
Description: DAVID results for triglycerides

File Name: Supplementary Data 4
Description: DAVID results for Rheumatoid arthritis

File Name: Supplementary Data 5
Description: Description of genes affected by lipid traits

File Name: Supplementary Data 6
Description: Weighted Median and weighted Mode-MR results for the 51 trait-gene associations found by revTWMR

File Name: Supplementary Data 7
Description: Weighted Median and weighted Mode-MR significant results missed by revTWMR

File Name: Supplementary Data 8
Description: DAVID results for RA (revTWMR + MR-mode and -median based significant genes)

File Name: Supplementary Data 9
Description: PheWAS results for rs2456973

File Name: Supplementary Data 10
Description: Gene perturbation and genetic correlations

File Name: Supplementary Data 11
Description: Correlation between observational correlation pvalues and gene-based test pvalues

File Name: Supplementary Data 12
Description: Proportion of observational correlation explained by TWMR, revTWMR and confounders

File Name: Supplementary Data 13
Description: Drug targets analysis

File Name: Supplementary Data 14
Description: Tissue-specific trait->gene associations

File Name: Supplementary Data 15
Description: Fisher test for enrichment of significant genes in mice data

File Name: Supplementary Data 16
Description: Summary Statistics of the GWASs
